# Treatment protocols for claw horn lesions and their impact on lameness recovery, pain sensitivity, and lesion severity in moderately lame primiparous dairy cows

**DOI:** 10.3389/fvets.2022.1060520

**Published:** 2022-12-08

**Authors:** Mohammed Babatunde Sadiq, Siti Zubaidah Ramanoon, Wan Mastura Shaik Mossadeq, Rozaihan Mansor, Sharifah Salmah Syed-Hussain

**Affiliations:** ^1^Department of Farm and Exotic Animal Medicine and Surgery, Faculty of Veterinary Medicine, Universiti Putra Malaysia, Serdang, Selangor, Malaysia; ^2^Department of Veterinary Clinical Studies, Faculty of Veterinary Medicine, Universiti Putra Malaysia, Serdang, Selangor, Malaysia; ^3^Department of Veterinary Pre-Clinical Science, Faculty of Veterinary Medicine, Universiti Putra Malaysia, Serdang, Selangor, Malaysia; ^4^Centre of Excellence (Ruminant), Faculty of Veterinary Medicine, Universiti Putra Malaysia, Serdang, Selangor, Malaysia

**Keywords:** lameness, claw lesions, hoof trimming, dairy cows, treatment, animal welfare

## Abstract

This study aims to investigate the effects of routine treatment protocols for claw horn disruptive lesions (CHDL) on lameness recovery rates, pain sensitivity, and lesion severity in moderately lame primiparous cows. A cohort of first parity cows was recruited from a single commercial dairy herd and randomly allocated to five treatments, comprising four lame groups (LTNB, LTN, LTB, and LT) and a single group non-lame group. Eligibility criteria for the lame cows included a first lameness score (score 3/5), presence of CHDL on a single foot, good body condition score of 3.0 to 3.5, and no history of previous lameness. LTNB received a combination of therapeutic trim, administration of a non-steroidal anti-inflammatory drug (NSAID; Ketoprofen) for 3 days, and hoof block on the healthy claw. Both LTN and LTB received the same treatment as LTNB without hoof block and NSAID, respectively. LT received only a therapeutic trim, whereas non-LT (negative control) received either a therapeutic or preventive trim. Pain sensitivity was assessed using the limb withdrawal reflex while lesion severity was recorded using the International Committee Animal Records (ICAR) Atlas guide. The enrolled cows were observed at weekly intervals, and the primary outcomes were assessed 28 days after treatment. The number (%) of recovered cows was 15 of 20 (75%), 13 of 21 (61.9%), 6 of 14 (42.9%), and 6 of 15 (40%) for LTNB, LTN, LTB, and LT, respectively. LTNB had significantly higher odds of successful treatment (OR = 4.5; 95% 1.1–19.1) compared to LT. Pain sensitivity based on limb withdrawal reflex was absent in a significantly higher number of cows (15/20; 75.0%) in LTNB compared to LTB and LT. LTB had a significantly lower lesion severity score in comparison to LTN. Overall, cows with limb withdrawal at day 28 after treatment were less likely (OR = 0.06; 95% CI 0.01–0.24) to develop a non-lame score. In conclusion, the treatment with therapeutic trim, hoof block, and NSAID led to better recovery and reduced pain sensitivity in moderately lame primiparous cows with good BCS compared to those that received only therapeutic trim. Further research on the changes within the hoof capsule following various treatment protocols is needed to elucidate the clinical benefits observed in this study.

## Introduction

Lameness is one of the most common health issues in dairy cattle, resulting in economic losses and severe impacts on animal welfare (1, 2). Claw horn lesions account for the majority of lameness events in dairy cows (3, 4). Lame cows are categorized either as mild, moderate, or severe depending on the extent of gait changes and postural defects (5), which may be associated with behavioral and production changes (6). For instance, severely lame cows spent less time at the feeding bunk (7), exhibited lower rumination time (8), loss in body condition (9), and produced less milk (2) compared to non-lame cows. Similar behavioral alterations were observed in newly and moderately lame cows (10), thereby highlighting the need for prompt detection and proper treatment. However, there is data paucity on the management of moderately lame cows affected with CHDL with a higher likelihood of dairy farmers underestimating lameness prevalence in such groups of cows (11, 12).

Claw horn disruptive lesions (CHDL) such as sole ulcer, sole hemorrhage, and white line disease are the most common conditions causing lameness in dairy cows (4, 13). Nevertheless, there is a deficit of information regarding the management of CHDL (14) with recent studies recommending further research to develop effective treatment protocols for these lesions (15, 16). Moreover, reports from previous studies highlighted that most information on the treatment of CHDL is based on experience and knowledge gained by field experts rather than evidence-based clinical trials (1, 14, 15).

A few studies have reported promising outcomes in the treatment of CHDL by a combination of therapeutic hoof trimming, application of hoof block to relieve pressure on the affected claw, and pain management using a non-steroidal anti-inflammatory drug (NSAID), such as Ketoprofen (15, 17). Higher recovery rates were observed when lame cows were promptly treated, whereas chronically lame cows failed to respond positively to the same treatment protocol (18). To elucidate the effects of various treatment protocols for CHDL, it is important to consider diverse lameness recovery measures, such as gait changes, lesion progression, and pain sensitivity.

Cow-level factors, such as parity, body condition score (BCS), previous lameness history, and lesion severity have also been demonstrated to influence the recovery rates of lame cows after treatment (3, 19). These factors need to be considered when evaluating the impact of a treatment protocol. For instance, parity has been widely reported as a risk factor for CHDL in dairy cows (19, 20). The sudden introduction to a new environment and housing systems may contribute to the onset of CHDL in first parity cows immediately after calving (21). These events are important on farms that frequently purchase young stock and lack routine hoof care management. This study aims to evaluate treatment options for moderate lameness caused by CHDL and their impacts on lameness recovery measures in first parity cows with good BCS. It was hypothesized that lameness recovery rates, gait changes and locomotion scores, pain sensitivity and lesion progression would vary with the treatment provided against CHDL in moderately lame cows.

## Materials and methods

### Study design

A randomized clinical trial (RCT) was applied in this study. Specifically, a positive controlled trial was conducted since all the enrolled animals were assigned to specific treatment groups. The protocol was reviewed and approved by the Institutional Committee for Animal Use and Care, University of Putra Malaysia (Ref: UPM/IACUC/ AUP-R010/2019) and this manuscript was prepared based on the guidelines outlined in the REFLECT statement for reporting RCT in livestock (22).

### Study herd (animal housing, feeding, and management)

This study was conducted on a dairy farm located in Linggi, Negeri Sembilan, Malaysia. The farm was one of the herds enrolled in a cross-sectional study to investigate the prevalence of lameness and associated risk factors in dairy farms in Peninsular Malaysia. The farm was considered suitable for the RCT based on its relatively large herd size (> 300 lactating cows), moderate lameness prevalence (20–29%), availability of trim chute and hoof care unit, and computerized health and production recording system. The farm manager's contact and email address were retrieved from the registry list provided by the Department of Veterinary Services, Putrajaya, Malaysia. Thereafter, the farm manager was briefed about the study objectives, inclusion criteria, and consent to participate.

The farm had a total of 452 Holstein Friesian and Friesian Sahiwal primiparous cows with an average milk yield of 4,575 L that were housed all through the year. The animals were kept in free stalls divided into four pens. Each stall has in-placed rubber mats, which were installed 3 years prior to this study. The floor at the walkway and feed alley was made up of concrete, whereas rubber mats were placed in the holding barn and milking parlor. A herringbone was used in milking the cows twice daily (6.00 am and 5:30 pm). Routine hoof trimming was performed weekly for cows in early (within 100 days in milk; DIM) and late lactation (above 200 DIM) by two farm staff using the five-step Dutch method. Hoof health data on the type and severity of claw lesions, claws affected, and the procedure performed was recorded electronically for each cow. Lame cows were identified by weekly locomotion scoring as per routine management and treated immediately upon detection. Other lameness management includes a footbath containing copper sulfate located at the exit of the milking parlor. The footbath was changed weekly by dissolving 200 grams of copper sulfate in 2 l of water. A motorized scrapper and manual water pumping systems were used to clean the floors at the milking parlor, resting barn, walkway, and holding yards. The cows were fed a total mixed ration composed of alfalfa hay, soyabean cake, fish meal, grain corn, and other supplements, which were adjusted depending on the stages of lactation.

### Animals selection and enrollment criteria

Cow selection and enrollment began in June 2020 until December 2020. Locomotion scoring was conducted for all the lactating primiparous cows in the herd by a trained observer twice a week as cows exited the milking parlor on a leveled and non-slippery surface. A five-point scoring (23) was applied and cows with locomotion scores of 1 (sound), 2 (mildly lame), and 3 (moderately lame) were selected. Acceptable intra-observer reliability was obtained (*Kp* = 0.88) upon comparing the locomotion scores of cows in two different pens on two occasions.

The inclusion criteria comprised cows presenting the first lameness event on a single hind or forelimb that were selected based on available farm records and a lame score (locomotion score equal to or >3) after two successive non-lame scores. Upon further screening, only those affected with CHDL (sole hemorrhage and ulcer; SHU and white line disease; WLD) on a single claw and having a good BCS (a score of 3.0–3.5 on a scale of 5.0) were enrolled. Meanwhile, cows with a history of treatment for lameness on any limb or had received parenteral antibiotics or NSAID within the previous 2 weeks, having low BCS (< 3.0), and within 10 DIM were not enrolled. Specifically, BCS was recorded using the five-point scoring scale employed by Vasseur et al. (24). The eligible cows were then restrained in a hoof-trimming chute and their hooves were examined. Detection of the lame foot entailed information from visual observation during locomotion scoring and the presence of withdrawal reflex upon applying pressure to the claw zones using a hoof tester. For the non-lame group, cows were considered eligible if they exhibited sound locomotion scores for at least 2 weeks before enrollment. Since the freestall was partitioned into four pens, an approximately equal number of cows were allocated to the various experimental groups from each pen.

Table 1 depicts the classification and description of the CHDL and corresponding severity scores recorded during the hoof examination as described by Sogstad et al. (25). Claw lesions were diagnosed by the researcher, a trained veterinarian, by using the ICAR claw health classification as a guide. Intra-observer reliability was not performed for claw lesion classification. Cows were enrolled dynamically as they fulfiled the inclusion criteria.

**Table 1:** Definition and categories of severity scores for claw lesions.

**Lesion**	**Score**	**Definition**
Sole ulcer	1	Corium is exposed, but unaffected
	2	Presence of granulation tissue, purulent exudates, necrosis and separation of the sole horn
	3	Involvement of the deeper structures of the claw
Sole hemorrhage	1	Mild haemorrhagic discolouration
	2	Moderate hemorrhage on a single spot and covering >20% of the sole surface (circumscribed)
	3	Marked hemorrhage on a single spot or extensive haemorrhagic discolouration covering >50% of the sole (diffused)
White line fissure	1	A fissure that disappears with a deep cut beneath the normal trimming level
	2	Deep fissure perforating next to the corium of sole or wall
	3	Corium is affected by purulent exudates, eventually with necrosis, granulation tissue, and separation of the wall or sole
White line hemorrhage	1	Slight haemorrhagic discolouration
	2	Moderate hemorrhage on a single spot or several superficial hemorrhages covering >20% of the white line
	3	Profound hemorrhage on a single spot or extensive haemorrhagic discolouration covering >50% of the white line

Adopted from Sogstad et al. (25).

### Random allocation and treatments administered

Enrolled animals were randomly allocated to five experimental groups (Table 2). Randomization was blocked with lesion type to ensure the matching of an equal proportion of cows with each diagnosis. The standard treatment protocol involved therapeutic hoof trimming, placing a hoof block on the healthy claw, and administration of NSAID (Ketoprofen) for 3 days.

**Table 2:** Experimental groups and treatments administered in the randomized clinical trial.

**Groups and animals enrolled**	**Treatment**	**Description**
LTNB (*n* = 24)	Standard treatment protocol	The standard treatment protocol consists of corrective trim, administration of NSAID, and application of hoof block on the healthy claw
LTN (*n* = 23)	Therapeutic trim + hoof block	Therapeutic trim plus hoof block attached to the healthy claw and no Ketoprofen administered
LTB (*n* = 16)	Therapeutic trim + NSAID	Therapeutic trim plus Ketoprofen, without hoof block, applied to the healthy claw
LT (*n* = 18)	Therapeutic trim only	Only therapeutic trimming
Non-LT (*n* = 15)	Only preventive or therapeutic trimming	Only preventive or therapeutic trimming

NSAID, non-steroidal and anti-inflammatory drug; STP, standard treatment protocol.

Therapeutic trim of the whole foot consisted of a standard application of the five-step Dutch method, trimming of the identified lesion, removal of the diseased horn, and ensuring a balance of the heel height and sole thickness for even weight distribution between the medial and lateral claws (15, 26). The hoof block comprised a natural wooden type (Vettec Animal Health, the Netherlands), approximately 110 mm long, 55 mm wide, and 23 mm deep, that was positioned on the healthy claw to replicate proper claw placement and weight distribution. An adhesive glue designed for bovine hooves (Bovi-BondTM 210 cc, Vettec Animal Health) was applied to facilitate the adhesion of the block to the claw. Meanwhile, the NSAID comprised a three-days course of ketoprofen (100 mg/mL) administered by deep intramuscular injection at 3 mg/kg.

LTNB received the standard treatment protocol; therapeutic trim, NSAID, and hoof block, LTN received therapeutic trim and hoof block, LTB received therapeutic trim and NSAID, and LT received only therapeutic trim. Meanwhile, the non-lame group (Non-LT) received either a preventive trim or therapeutic trim with an emphasis on reducing the overgrown claw, debriding the claw lesion, and ensuring a balanced sole surface between the medial and lateral claws as described in our previous study (26). Preventive hoof trimming entailed the use of the five-step Dutch method without completing the final step, which is the removal of a loose horn. All treatments were performed by a trained veterinary surgeon (the first author of this manuscript) who is familiar with the management of lame cows. A farm staff assisted the researcher to restrain the cows during treatment and was responsible for the administration of NSAID when the researcher was absent. Restraint and treatment durations were recorded in minutes.

Enrolled animals were identified using tag numbers and body marking for easy identification from a distance. Cows were managed according to the farm management, and farm staff were informed to notify the researcher if any further treatment is necessary. Under such situations, the cows were retreated as per the treatment groups by the trained farm staff or the researcher if visiting the farm within 24 h of receiving the complaint. The farm staff was aware of the different treatment protocols used in the study and the specific cows treated in each group.

### Limb withdrawal, body movements, and vocalization

Limb withdrawal, body movements, and vocalization were recorded during treatment. The cows were initially allowed to remain standing in the trim chute for 5 min after being restrained. Upon immobilizing the affected limb, limb withdrawal was assessed using a hoof tester by exerting mechanical pressure on the suspected lesion site and claw zones. Limb withdrawal was considered positive when the cow exhibited mild twitches or attempted to adduct the restrained limb. Agitation or body movement was defined as the movement of both the treated limb and other body parts (neck and body) during treatment. Vocalization was defined as oral sound by the treated cow as a sign of discomfort. The outcomes were considered dichotomous and recorded as either present or absent.

### Treatment follow-up and outcome measures

A 28-days observation period was adopted in this study. Locomotion scores, lesion severity, and pain sensitivity were recorded for each cow during treatment (day 1) and on days 7, 14, 21, and 28. The first follow-up observation was on 7 days (±3 days) after treatment as cows were assessed for locomotion scores and inspection of the lesion sites. The same locomotion and lesion-scoring techniques described earlier were employed to monitor the lameness recovery rate and lesion progression, respectively. Pain sensitivity was recorded using limb withdrawal and recorded as a dichotomous outcome (present or absent). Both vocalization and agitation or body movements were also recorded during the follow-up periods.

For LTNB and LTN, the block was re-applied if absent at any observation points. Animals were re-treated if their locomotion score had deteriorated from the time of enrollment to 14 or 21 days (±3 days) post-treatment. No cow was given additional NSAID therapy beyond 3 days. The final examination was on 28 days (±3 days) after treatment. At this stage, the hoof block was removed using a hoof nipper and careful leverage. Cows that were sold, culled, or died before the measurement of the primary outcomes were identified and removed from the study. All study outcomes were observed by the trained veterinarian and blinding was not applied.

### Study inclusions

Primiparous cows were selected and assessed from June 1, 2020, to November 30, 2020. Cows' enrollment was discontinued on December 1, 2020, due to the low number of animals available for recruitment, an increased workload and few available staff to assist the researchers due to the introduction of new pregnant heifers. During the study period, 26 lame animals were not enrolled due to the following reasons: eight had severe hock lesions; six were lame but showed no visible claw lesion, two had digital dermatitis, six had swelling of the coronet, and four had either mastitis and downer cow syndrome that affected their locomotion. Meanwhile, five sound cows from the non-LT were excluded due to treatment for other post-calving complications aside from lameness.

Overall, a total of 96 cows were eligible for enrollment upon fulfilling the inclusion criteria (LTNB = 24, LTN = 23, LTB = 16, LT = 18, and non-LT = 15) but 11 of them were withdrawn before completing the study period. **Table 5** outlines the allocation of cows (*n* = 85) to each treatment according to claw lesion diagnosis. A total of 51 cows (50.5%) were treated for sole ulcer, 20 (19.8%) for white line disease, and 14 (13.8%) for sole hemorrhage (Table 3).

**Table 3:** Number of cows allocated to each treatment based on claw lesion diagnosis and overall cows that completed the trial.

**Claw lesion diagnosis**					
**Groups**	**Overgrown hoof**	**Sole hemorrhage**	**Sole ulcer**	**White line disease**	**At 28-d post-enrollment**
LTNB	3 (16)	2 (16)	14 (62.5)	5 (20.8)	20
LTN	4 (21.7)	2 (13.0)	13 (60.8)	4 (17.4)	21
LTB	2 (18.7)	2 (18.7)	10 (62.5)	3 (18.7)	14
LT	2 (16.7)	3 (16.7)	11 (61.1)	4 (22.2)	15
Non-LT	5 (33.3)	5 (33.3)	1 (6.7)	4 (26.7)	15
Total (%)	16 (15.8)	14 (13.8)	51 (50.5)	20 (19.8)	85

Some lame cows had both overgrown hooves and CHDL. All cows were enrolled for having a single claw horn lesion, either sole hemorrhage, sole ulcer, or white line disease. Overgrown hooves were not considered claw horn lesions.

### Excluded cows

A total of 11 cows were withdrawn or lost before the 28-days study period. Four cows were culled (LT = 2 and LTNB = 2), 2 died (LT = 1 and LTB = 1), three were treated for other illnesses aside from lameness (LTNB = 1 and LTN = 2), and two were withdrawn for non-compliance with the study protocol after enrollment (LTNB = 1 and LTB = 1). No cow was removed from the NL group. Of the 70 cows that completed the RCT, eight of them required re-treatment at the 7 d (±3) after enrollment. Specifically, three cows received additional therapeutic trimming (LTNB = 1 and LTB = 2), seven cows had removed their hoof block, which was re-applied (LTNB = 3 and LTN = 4), and four cows were treated for hock lesions by wound cleaning and application of oxytetracycline spray (Woundsarex^®^).

### Data analysis

Data analysis was conducted using the Statistical Package for Social Science (SPSS, IBM Version 23.0). Descriptive statistics were used to summarize the characteristics of the enrolled cows. To determine the success of random allocation, a one-way analysis of variance (ANOVA) was applied to evaluate differences in DIM, milk yield, lesion severity, time spent to restrain the cows, and treatment time between the groups during enrollment. Cross-tabulation was used to compare the categorical variables, such as vocalization, agitation, and limb withdrawal between the groups during enrollment and other follow-up periods. The outcome was defined as non-lame (locomotion score < 3) or lame (locomotion score 3 and above) at each observation point after treatment. Final comparisons between treatments were based on the primary outcomes recorded at 28-days post-treatment (recovery rate, pain sensitivity, and lesion severity).

The proportions of recovered cows were compared between the treatments using cross-tabulation. A non-parametric test, the Kruskal Wallis test, was utilized to compare the pain sensitivity and lesion severity between the treatments since the data were not normally distributed. Logistic regression models were built to test for the association between covariates and successful treatment at 28 days post-treatment. The variables considered were the location of treated limbs (left and right hind limb), breed, BCS during treatment [3 and 3.5], DIM during enrollment, lesion type; sole hemorrhage/ulcer and white line disease, lesion severity, re-treatment at any observation point [7 and 14 days after treatment; Yes or no], and treatments. A two-stage model building process: univariable and multivariable model. At the univariable level, *P* < 0.1 was considered for factors to be introduced into the multivariable model. A forward conditional method was applied in the final model and *P* < 0.05 was considered for significant differences. Treatment was forced into the models as categorical fixed effects and model fit was evaluated based on the change in the Akaike information criterion upon removing a covariate.

## Results

### Descriptive results and univariate analysis

Table 4 presents the characteristics of the enrolled cows. Overall, the median (IQR) LS was 3 (0) while the mean (± SD) DIM and daily milk yield during enrollment were 68.9 (± 6.5) and 12.9 (± 2.4), respectively. The mean (± SD) time taken to restrain and treat the cows was 7.1 (± 2.6) and 12.2 (± 3.4) min, respectively. Most of the cows (88.3%) were treated for claw lesions on the rear foot. There was no significant difference in BCS, lesion severity and lameness scores at treatment, treated foot, and the mean milk yield between all the treatments. All cows were enrolled at early to mid-lactation (within 220 DIM), but the mean DIM for LT and non-LT were significantly higher (P < 0.05) compared to LTNB, LTN, and LTB. The time taken to restrain cows was not significantly different between the treatments; however, LT and non-LT recorded the highest and lowest mean treatment time (*P* < 0.05), respectively compared to LTNB, LTN, and LTB. Vocalization was absent in a higher proportion of cows during treatment (78.8%; 67/85), whereas 68.4% (59/85), and 76.6% (65/85) showed agitation and limb withdrawal, respectively. The proportion of cows with vocalization and agitation was not different between treatments, but Non-LT recorded a significantly lower number of cows (*P* < 0.05) with limb withdrawal compared to other treatments.

**Table 4:** Descriptive statistics of primiparous cows enrolled in the five experimental groups.

**Variables**	**LTNB**	**LTN**	**LTB**	**LT**	**Non-LT**	**Overall**
No. of animals	20	21	14	15	15	85
Median locomotion score	3	3	3	3	1	3
Lesion severity (Mean ± SD)	3.2 ± 0.5	3.4 ± 0.6	2.9 ± 0.9	3.3 ± 0.8	2.1 ± 0.7	3.2 ± 0.7
DIM at enrolment (Mean ± SD)^a^	53.1 ± 8.89	50.0 ± 7.71	55.7 ± 15.7	83.5 ± 22.4	114 ± 15.6	68.9 ± 6.5
BCS at enrolment (Median [IQR])	3 (0)	3 (0)	3 (0)	3 (0)	3.5 (0)	3(0)
Last recorded milk yield/day (kg; Mean ± SD)	17.4 ± 0.5	18.2 ± 0.5	17.5 ± 0.6	12.9 ± 0.6	12.6 ± 0.6	15.7 ± 0.3
Restraint duration (min; Mean ± SD)	7.6 ± 3.2	6.0 ± 1.3	7.5 ± 3.1	7.3 ± 2.4	7.3 ± 2.6	7.1 ± 2.6
Treatment duration (min; Mean ± SD)^a^	12.9 ± 2.1	12.4 ± 3.2	11.7 ± 2.4	15.3 ± 2.7	8.4 ± 0.7	12.2 ± 3.4
**Treated limb/location of the lesion**						
Rear left (%)	8	10	6	5	6	35 (41.2)
Rear right (%)	10	8	5	8	9	40 (47.1)
Front left (%)	1	0	1	0	0	2 (2.3)
Front right (%)	1	3	2	2	0	8 (9.4)
Vocalization during treatment (%)	3	5	4	4	2	18 (21.1)
Agitation during treatment (%)	17	12	10	12	8	69 (81.1)
Limb withdrawal during treatment^a^ (%)	18	19	12	15	1	66 (77.6)

a Difference between experimental groups was significant.

### Recovery rate

Table 5 presents the locomotion scores of the enrolled cows on day 28 after treatment. The number (%) of recovered cows was 15 of 20 (75%) for LTNB, 13 of 21 (61.9%) for LTN, 6 of 14 (42.9%) for LTB, and 6 of 15 (40%) for LT at 28-days after treatment. The highest proportion of recovery rates was recorded at 14 (40.0%; 16/40) and 21 d (35.0%; 14/40) after treatment, with the majority in LTN (50%; 8/16) and LTNB (42.8%; 6/14) at 14 and 21-days post-treatment, respectively. LTNB had higher odds of successful treatment at 28 d after treatment (OR = 4.5; 95% 1.1–19.1) compared to LT, but no significant difference was detected between the latter and other groups.

**Table 5:** Number of recovered cows at various follow-up periods in each treatment and the final recovery rate at 28 days after treatment.

**Follow-up period and number of recovered cows**						
**Groups**	**Day 7**	**Day 14**	**Day 21**	**Day 28**	**Recovered (%)**	**Still lame (%)**
LTNB^ab^	2	6	6	1	15 (75.0)	5 (25.0)
LTN ^ac^	1	8	3	1	13 (61.9)	8 (38.1)
LTB^c^	0	0	3	3	6 (42.9)	8 (57.1)
LT^c^	1	2	2	1	6 (40.0)	9 (60.0)
Total (%)	4 (5.7)	16 (22.9)	14 (20.0)	6 (8.6)	40 (51.2)	30 (42.8)

Cows with successive non-lame scores after treatment were considered to have recovered and such cows were not re-introduced to the study if they became lame afterwards. Groups with different superscript letters are significantly different.

### Pain sensitivity and lesion severity

Pain sensitivity based on limb withdrawal reflex was absent in a significantly higher number of cows (15/20; 75.0%) in LTNB compared to LTB (35.7%) and LT (40.0%) on day 28 after treatment (Table 6). Meanwhile, no significant difference was detected in the number of cows with limb withdrawal reflexes between LTN, LTB, and non-LT. LTN had the highest lesion severity score, followed by LT, LTNB, and LTB. Statistical difference was only detected between LTN and LTB. We observed no correlation between limb withdrawal reflex and lesion severity score. Specifically, the lowest lesion severity score was recorded in the group (i.e., LTB) with the highest percentage of animals with limb withdrawal present.

**Table 6:** Lesion severity score (Mean ± SD) and number of cows with and without limb withdrawal reflex.

**Groups**	**Limb withdrawal** **reflex on day 28**		**Lesion severity score** **at day 28 (Mean ±SD)**
	Absent (%)	Present (%)	
LTNB	15 (75.0)^a^	5 (25.0%)	2.15 ± 0.48^ab^
LTN	11 (52.4)^ab^	10 (47.6%)	2.38 ± 0.49^a^
LTB	5 (35.7)^b^	9 (64.3)	1.86 ± 0.66^b^
LT	6 (40.0)^b^	9 (60.0)	2.20 ± 0.67^ab^
Total (%)	37 (52.9)	33 (47.1)	2.17 ± 0.58

The limb withdrawal reflex on day 28 after treatment was compared between the groups using cross-tabulation and Chi-square test. Non-LT was not included since claw horn lesions were not present in all the cows during enrollment. Groups with different superscripts are significantly different at *p*-value = 0.05. Comparisons are along the column for each variable.

### Logistic regression for factors associated with recovery rate

Table 6 shows the results of the multivariable model. Cows with limb withdrawal at d 28 post-treatment were less likely to recover relative to those without limb withdrawal. Treated limb (rear left or rear right limb) and restrain time during enrollment were not associated with recovery rates. However, there was a trend for lower odds of recovery among cows with vocalization at d 28 after treatment compared to those without vocalization (Table 7).

**Table 7:** Final logistic regression model showing the odds ratios of lameness recovery between the treatment groups and other associated factors at day 28 after treatment.

	**B**	**S.E**.	**Wald**	***P*-value**	**OR**	**95% CI**
**Groups**			5.51	0.04		
LTNB	1.50	0.73	4.15	0.02	4.50	1.09–19.1
LTN	0.89	0.69	1.65	0.19	2.43	0.62–9.47
LTB	0.11	0.75	0.02	0.87	1.12	0.25–4.93
LT					Ref	
**Treated foot**						
Rear right limb	-0.95	0.63	2.24	0.13	0.38	0.11–1.34
Rear left limb					Ref	
**Limb withdrawal**						
Present	-2.78	0.70	15.67	0.001	0.06	0.01–0.24
Absent						
**Restrain duration**	0.14	0.12	1.22	0.26	1.15	0.89–1.47
**Vocalization**						
Present	-1.31	0.78	2.79	0.09	0.26	0.05–1.25
Absent						

CI, confidence interval, OR, odds ratio, SE, standard error, Ref, reference group.

## Discussion

Depending on the treatment administered, the recovery rates in moderately lame primiparous cows with good BCS treated for CHDL on a single foot were recorded in the present study. Cows that received therapeutic trim, NSAID, and hoof block (LTNB) recorded a significantly higher recovery rate than cows treated with only therapeutic trim (LT), which is consistent with previous findings (15, 16). The treatment protocol received by LTNB has been advocated for the treatment of CHDL as it corresponded to a shorter time for the restoration of normal gait in lame cows (15, 27). Wilson et al. (27) in a recent RCT also revealed that cows treated as in LTNB were less likely to become lame or severely lame (OR = 0.66 and 0.28) compared to those that received therapeutic trim and hoof block only when deemed necessary. However, non-lame cows were recruited in the latter study and observed until the first lameness event either before or after calving.

The primary outcome measures in this study were lameness score, nociceptive response (pain sensitivity), and lesion severity score on day 28 after treatment. Apart from these events, the other mechanisms underlying the causal effect of the standard treatment protocol (therapeutic trim, hoof block, and three-day course of NSAID) could not be elucidated in this study. There are several mechanisms through which the positive effects could occur. The underlying events in the pathogenesis of CHDL are the compression of the sole corium and subsequent vascular dysfunction leading to ischemia and hemorrhage (19, 27). The blocking of the healthy claw reduces the load-bearing and compression of the corium in the affected claw, thereby promoting injury healing. Likewise, the administration of NSAIDs might elicit a direct effect on the corium by reducing systemic and local inflammation in the hoof capsule and promoting healing following reduced loading due to block application. These events might explain the higher cure rates in LTNB compared to LT which received only therapeutic trim.

The highest proportion of recovered cows was recorded at 14 (40.0%; 16/40) and 21 35.0%; 14/40) days after treatment, especially in LTN (50%; 8/16) and LTNB (42.8%; 6/14). The inflammation associated with CHDL has been found to stimulate exostosis development (20) and digital cushion adipose metabolism (19, 27), especially a few weeks after the onset of the primary lesion. The implementation of early detection and treatment in the present study might have prevented the aforementioned lesion progression while contributing to high cure rates as demonstrated in prior research (18, 28, 29). Overall, the recovery rates in this study ranged from 45 to 70% which is higher than the reports by Thomas et al. (15). Differences in lameness definition, lesion severity, and observation period may contribute to the disparity. The high cure rates in our study could be linked to the relatively smaller dataset and less diversity in farm management compared to those of Thomas et al. (15). The fact that all the enrolled cows were in first parity, moderately lame, and experiencing their first lameness events may also contribute to the higher cure rates in our study.

Higher proportions of recovered cows were observed in LTNB compared to LTN and LTB but the difference was not statistically significant. LTNB and LTN were treated with a combination of therapeutic trim and blocking the healthy claw, while the latter group was not administered NSAID. Likewise, the mall number of cows in each group may explain this finding. The result reflects that blocking the healthy claw might play a more critical role in facilitating a faster healing process and improving the locomotion score. Furthermore, the finding aligns with that of Thomas et al. (15), where marginal differences in cure rates occurred between cows treated with NSAID without block and vice versa. Given that blocking the healthy claw is vital in reducing the compression of the corium and pressure on the affected claw, the non-significant difference between LTN and LTB highlights the multifaceted events leading to pain and gait disturbance in moderately lame cows affected with CHDL.

Most of the enrolled cows were moderately lame with less severe claw lesions. Hence, a significant difference might not be reflected in the gait scores when either the hoof block or NSAID is missing from any of the treatments employed in this study. On the other hand, severe lesions may respond differently to treatment involving block and NSAID as both stages of corium compression and end-stage inflammation are present. For instance, block application in lame cows led to improved gait properties but the difference in weight distribution across the limbs was smaller in cows with more severe lameness than in mildly lame cows (30). Further investigations comparing treatment protocols in lame cows affected with claw lesions of varying severity would assist in elucidating the underlying events.

Notably, a few cows in LTNB, LTN, LTB, and LT developed lameness on the contralateral hind limb on day 28 after treatment. Two cows from Non-LT also developed lameness on the trimmed foot and contralateral limb during the study period. Block application may provoke discomfort and behavioral alterations that may initiate redistribution of weight-bearing between the claws (30), thereby resulting in a lower healing rate of the affected claw (15). However, concurrent development of claw lesions might occur in both hind limbs and subsequent onset of lameness at different periods. Further work is required to elucidate these findings, especially the varying loadings on the contralateral hind limb following blocking on the healthy claw adjacent to the diseased counterpart.

Limb withdrawal reflex was also assessed in this study as an indicator of lameness recovery. A significantly higher number of cows in LTNB did not exhibit this behavior on day 28 after treatment compared to LTB and LT. Further analysis also revealed a correlation between limb withdrawal and locomotion scores at the end of the trial. Limb withdrawal employed in this study is similar to the assessment of pressure or mechanical nociceptive response (31–34) and leg movements (17) in previous related research involving CHDL. The result highlights that limb withdrawal can be used for the detection of CHDL, degree of lameness, pain assessment, and monitoring recovery after treatment. A similar result was reported by Passos et al. (33) in which positive associations were found between the nociceptive response and locomotion of cows affected with sole ulcers and white-line disease. Likewise, a prior study documented that severely lame cows exhibited significantly higher frequencies of limb withdrawal upon attempting to rotate or compress the affected claw with a hoof tester (35). The present findings add to the existing body of knowledge that such behavior could be employed to monitor the recovery of lame cows, especially those suffering from acute CHDL.

In terms of lesion severity score, animals in LTB that were treated with therapeutic trim and hoof block without NSAID recorded the lowest lesion severity score, which was significantly different relative to LTN. Although this result may support the earlier discussion that blocking has positive effects on the corium and promotes injury healing, the insignificant finding compared to LT contradicts such an event. Overall, this finding reflects that lesion severity score may not be a good indicator of lesion progression, particularly for moderately lame cows suffering from CHL, which is consistent with reports from previous studies (17, 35). Another important result is the lack of correlation between the presence of limb withdrawal and lesion severity scores 28 days after treatment. Notably, LTB recorded the lowest lesion severity score and the highest percentage of cows with limb withdrawal present. Reports from previous studies distinguishing between lameness and CHL may explain our finding. Groenevelt et al. (29) found that some cows with severe lesions such as sole ulcers and toe necrosis were only moderately lame. In another study, a positive correlation was observed between severe lesions and greater perturbed locomotion, but some cows with normal gait appeared to have severe lesions (35, 36). Thus, the visual appearance of CHL either during hoof examination (i.e., during diagnosis) or after treatment may not depict the actual pain responsible for gait changes in lame cows. Meanwhile, the limb withdrawal reflex correlates with changes in locomotion scores and a pointer of lameness recovery after treatment as observed in this study. Since lameness is indicated by abnormal locomotion, particularly derangements in gait symmetry, limb withdrawal is more likely to reflect such changes rather than those observed visually on the claw.

The difference in lameness recovery rates between LTNB and LT and the association with limb withdrawal was further confirmed in the logistic regression model. Specifically, LTNB had four times higher odds of successful treatment compared to LT, which is consistent with previous studies as discussed earlier (15, 30). There was also a tendency for lower recovery rates in cows that exhibited vocalization during the last observation period. Vocalization has not been widely studied in lame dairy cows. A recent study reported that both non-lame and acutely lame cows affected with CHDL expressed vocalization during restraining (17). This result requires further investigation, as it may be a sign of hyperalgesia in chronically lame cows or hyperactivity due to discomfort during restraint and lifting the foot for examination.

Our findings add to the existing literature on the treatment of CHDL in dairy cows. The randomization of the recruited cows and the primary outcomes considered also signify the strengths of this study. The presence of a non-lame group (Non-LT) allowed for the comparison with other treatments during the follow-up period. This study was designed using an RCT performed per best practice standards (REFLECT guidelines), indicating that outcomes were unlikely to be influenced by bias. RCT provide strong evidence of a causal effect (37) and the intervention had no negative effects on health and welfare parameters in the various treatments.

However, certain limitations in this RCT could be considered in future studies. For instance, only primiparous cows with good BCS and affected with CHDL were enrolled, hence the findings might not be generalisable to cows with dissimilar characteristics. Conducting this experiment on a single farm and the small number of cows in each treatment also reflects a weakness in this study. These limitations might have affected the power and the chances of detecting a significant difference in the primary outcomes. This study was time-consuming, and expensive and was conducted for 7 months, which denotes why only a few RCTs on the management of lame cows have been published. We did not consider the influence of season on the primary outcomes, since all the cows were enrolled between June and December 2020, which is regarded as the dry season in Malaysia.

## Conclusion

In conclusion, our findings revealed that treatment with therapeutic trim, blocking, and NSAID led to better recovery and reduced pain sensitivity in moderately lame primiparous cows with good BCS compared to those that received only therapeutic trim. These positive effects might not be reflected in the subjective lesion severity assessment. Hence, there are welfare benefits when lame cows are promptly detected and treated using a combination of therapeutic trim, hoof block, and pain management. Further research on the changes within the hoof capsule following various treatment protocols is needed to elucidate the underlying mechanisms in the clinical benefits observed in this study.

## Data availability statement

The raw data supporting the conclusions of this article will be made available by the authors, without undue reservation.

## Ethics statement

The animal study was reviewed and approved by the Institution of Animal Care and Use Committee, Universiti Putra Malaysia (Ref.UPM/IACUC/AUP-R010/2019). Written informed consent was obtained from the owners for the participation of their animals in this study.

## Author contributions

SR, WS, and MS contributed to the conception of the research and funding. SR and MS made substantial contributions to data acquisition, analysis, interpretation, drafted and revised the work, and wrote the final version. SS-H and RM made substantial contributions to revising the drafted manuscript. All authors approved the final version of the article and agreed to be accountable for all aspects of the work.
